# Analysis of the β-Glucosidase Family Reveals Genes Involved in the Lignification of Stone Cells in Chinese White Pear (*Pyrus bretschneideri* Rehd.)

**DOI:** 10.3389/fpls.2022.852001

**Published:** 2022-05-10

**Authors:** Han Wang, Yingjie Zhang, Xiaofeng Feng, Fulei Peng, Muhammad Aamir Mazoor, Yang Zhang, Yu Zhao, WenLong Han, Jinjin Lu, Yunpeng Cao, Yongping Cai

**Affiliations:** ^1^School of Life Sciences, Anhui Agricultural University, Hefei, China; ^2^CAS Key Laboratory of Plant Germplasm Enhancement and Specialty Agriculture, Wuhan Botanical Garden, Chinese Academy of Sciences, Wuhan, China

**Keywords:** pear, β-glucosidase, gene function, lignin synthesis, stone cells

## Abstract

BGLU β-glucosidases in glycoside hydrolase family 1 (GH1) are involved in many processes of plant secondary metabolism. In particular, its de-glycosylation function plays an important role in the transport of lignin monolignols. No comprehensive study of the BGLU family in Chinese pear (*Pyrus bretschneideri* Rehd.) has been reported yet. In this study, the 50 BGLU family members from Chinese white pear were identified. Three candidate genes, *PbBGLU1*, *PbBGLU15*, and *PbBGLU16*, that may be involved in lignin synthesis were screened by bioinformatics analysis and qRT-PCR. Subcellular localization showed that all three of these candidate genes were expressed in the extracellular region. Then, we analyzed the functions of *PbBGLU1* and *PbBGLU16. In situ* hybridization analysis showed that *PbBGLU1* transcripts were not only localized to some pulp cell walls, lignin deposition, and stone cell areas of a pear fruit, but that was also a small amount of enrichment in normal pear flesh cells. *PbBGLU16* transcripts were only enriched in lignin deposition and stone cell areas of pear fruit. Enzyme activity analysis revealed that GST-PbBGLU1 and GST-PbBGLU16 had a stronger activity and higher catalytic efficiency for coniferin than syringin. In addition, GST-PbBGLU16 exhibited the higher activity and catalytic efficiency for the two substrates compared with GST-PbBGLU1. The transformation of *PbBGLU1* and *PbBGLU16* into *Arabidopsis* identified that the lignin contents of *Arabidopsis BGLU-45* mutant, *PbBGLU1-RE*, and *PbBGLU16-RE* were not changed than that of wild-type. However, compared with wild-type *Arabidopsis*, the overexpression of the plant’s lignin increased in varying degrees. The effect of *PbBGLU16* on the lignin increment was greater than that of *PbBGLU1* in *Arabidopsis*. In pear fruits, with transient overexpression of *PbBGLU1*, the contents of lignin and stone cells were significantly higher (0.01 < *P* < 0.05) than those with empty vector injection pear fruits. After transient expression of *PbBGLU16*, lignin in pear fruit increased significantly (0.01 < *P* < 0.05) and stone cells showed a very significant difference (*P* < 0.01) compared with the control group. However, RNA interference silenced these two genes in pear fruit, which seemed to have no impression on lignin and stone cells. This study provides a molecular biological basis for improving pear fruit quality at the molecular level.

## Introduction

Pear belongs to the *Rosaceae* family and is one of the most important deciduous fruit tree species worldwide (Wu J. et al., 2012). “Dangshan Su” pear (*Pyrus bretschneideri* cv. Dangshan Su) is a diploid pear variety that originated in Dangshan County, Anhui Province, China. It not only has good flavor and high nutritional value but also has medicinal value and is very popular among people. However, due to its high content of stone cells, its economic value is not high enough (Yan et al., 2014). Stone cells are a kind of specific cell in pear fruit, and they are among the important factors that affect pear fruit processing and fresh consumption. Current research shows that the diameter, number, and density of stone cells have significant effects on pear fruit quality. The greater the diameter and density of the cell mass is, the greater the stone cell content of pear fruit, and the worse the fruit quality is (Zhang et al., 2017). Stone cells are formed by secondary thickening of parenchyma cells, and lignin is one of the crucial components of stone cells (Cheng et al., 2019a; Su et al., 2019).

Lignin is a secondary metabolite. Lignin synthesis, transportation, and deposition are closely related to stone cells development during pear fruit development (Cai et al., 2010; Jin et al., 2013). Structurally, lignin is a phenylpropanoid polymer mainly derived from three kinds of monolignols (*p*-coumaryl alcohol, coniferyl alcohol, and sinapyl alcohol) connected by different chemical bonds (Boerjan et al., 2003; Barros et al., 2015). Glycosylation of these three monolignols is catalyzed by UDP-glucosyltransferase (UGT) into monolignol glucosides (*p*-coumarin, coniferin, syringin) (Cheng et al., 2019b; Perkins et al., 2019), and glycosylation increases the solubility and stability of monolignols, which is beneficial to the transport and storage of monolignols (Gachon et al., 2005). Studies have found that a large number of monolignol glucosides can be stored in the vacuoles of different xylem cells (Steeves et al., 2001; Tsuji and Fukushima, 2004). In-depth research on the transport mechanism of monolignols is scarce, but the academic community currently believes that there are three transport mechanisms for the extracellular secretion of monolignols, namely, passive diffusion (PD), vesicle-related exocytosis, and the use of ABC transport factors or proton coupling to reverse the active transport of ATP (Miao and Liu, 2010; Alejandro et al., 2012; Barros et al., 2015). These monolignol glucosides are then transported to specific parts of the cell wall and hydrolyzed to lignin monolignols under the action of β-glucosidase, ultimately forming guaiacyl (G), syringyl (S), and p-hydroxyphenyl (H) units by laccase and peroxidase [dicotyledons have only guaiacyl (G) and syringyl (S) lignin] (Liu, 2012).

In recent years, an increasing number of species of the β-glucosidase family have been discovered, and the function of the β-glucosidase-encoding gene has become clearer. In 1994, coniferin-specific β-glucosidase was identified in the xylem of two Pinus species (Leinhos et al., 1994; Dharmawardhana et al., 1995). A study in 1999 found that coniferin β-glucosidase catalyzed the hydrolysis of monolignol glucosides to release cinnamyl alcohols for oxidative polymerization to lignin (Dharmawardhana et al., 1999). *At1g61810* (*AtBGLU45*), *At1g61820* (*AtBGLU46*), and *At4g21760* (*AtBGLU47*) in *Arabidopsis* play an important role in the synthesis of lignin (Escamilla-Treviño et al., 2006; Chapelle et al., 2012). *Os4BGlu14*, *Os4BGlu16*, and *Os4BGlu18*, which are members of the rice β-glucosidase family, have been proven to be functional genes involved in rice lignin synthesis (Baiya et al., 2018). In addition, a β-glucosidase was identified from poplar and named “PtrBGLU6”; this gene plays an important role in poplar lignification and cell wall formation (Tsuyama and Takabe, 2015).

In our study, all β-glucosidase family members in pear had been identified and further three β-glucosidase (*PbBGLU1*, *PbBGLU15*, and *PbBGLU16*) candidate genes were considered as functional genes for pear lignin synthesis. We through a series of experiments including *in situ* hybridization, *in vitro* enzyme activity studies, stable expression in *Arabidopsis thaliana* and transient expression in pear fruit. We provided evidence that *PbBGLU1* and *PbBGLU16* can act in the formation of lignin and stone cell development in pear fruit.

## Materials and Methods

### Identification of *BGLU* Genes in Five *Rosaceae* Species

The Chinese white pear genome database was obtained from the Pear Genome Project.^1^ To identify putative BGLU genes from Chinese white pear, several approaches were employed. We downloaded the HMM profile (PF00232) from the Pfam website^2^ (Finn et al., 2010) and searched for candidate genes using the HMM with *E*-values < 1*e*-10 from the Chinese white pear genome (Zhou et al., 2016). After performing multiple sequence alignment, the retrieved domain sequences of the pear are aligned, and the HMM of the BGLU genes family of the pear is constructed again. Using the constructed new hidden Markov model with *E*-values < 1*e*-10 to blasted against the Chinese pear genome for carryout PbBGLU genes. The online site SMART^3^ (Letunic et al., 2012) was used to determine the domain PF00232 of BGLU. The online analysis tool EXPASY^4^ was used to predict and analyze the physical and chemical properties of the obtained BGLU amino acids sequences.

### Phylogenetic Conserved Motif and Exon-Intron Structures Analyses of BGLU Genes in Pear

Sequence alignment of *PbBGLUs* proteins was done by using the MUSCLE method in MEGA 7.0 software. The phylogenetic tree was constructed with MEGA 7.0 software using the neighbor-joining (NJ) (bootstrap = 2,500) (Kumar et al., 2016). The conservative motifs were analyzed by MEME^5^ software the maximum value of the motif was set to 20, and the motif length was set between 6 and 50 (Bailey et al., 2015). The Gene Structure Server^6^ was used to generate exon-intron maps (Hu et al., 2015).

Phylogenetic analysis of *BGLU* gene family in *Arabidopsis*, *P. bretschneideri, Oryza sative*, and *Populus*. The software MEGA 7.0 was used to construct the phylogenetic tree. The amino-acid sequences of *Arabidopsis*, *O. sative*, and *Populus* were obtained from phytozome database.^7^

### Plant Materials, Treatments, and Growth Conditions

The materials were from “Dangshan Su” pear (*P. bretschneideri* cv. Dangshan Su) with the same growing environment for 40 years in Dangshan County, Anhui Province, China. We collected the mature leaves, buds, stem segments, and flowers on March 30, 2021; and collected the fruit on April 15 (15 days after flowering), 2021; May 14 (39 DAF), 2021; May 22 (47 DAF), 2021; June 4 (55 DAF), 2021; June 12 (63 DAF), 2021; June 28 (79 DAF), 2021; and August 30 (145 DAF), 2021. Each sample was frozen in liquid nitrogen and brought back to the laboratory for use. All samples were stored at–80°C for subsequent RNA extraction.

*Arabidopsis thaliana* L. wild type was purchased from the American Arabidopsis Biological Resources Center. *Arabidopsis At1g61810 bglu45-2* mutant seeds corresponding to the Salk_117269 flanking sequence tag were ordered from the Salk Institute Genomic Analysis Laboratory. Homozygous *BGLU45-2* mutant plants were isolated by genotype analysis with *Arabidopsis* BGLU45 gene and T-DNA specific primers, as described by Chapelle et al. (2012). The *Arabidopsis* plants were cultivated in a growth chamber at 23°C under 16/8 h light/dark cycles (constant illumination 100 μEm-2s-1). Ten replicates of each line were planted, and three similarly growing plants were collected for further analysis.

### RNA Extraction, Reverse Transcription PCR, and Real-Time PCR Analysis

The total RNA of all the used “Dangshan Su” pear fruits in this study was extracted using a Plant RNA extraction kit from Tiangen (Beijing, China). Total RNA (1 μg) from each sample was used in reverse transcription. First-strand cDNA was synthesized with a PrimeScript™ RT reagent kit with a gDNA Eraser kit (TaKaRa, Kyoto, Japan). All RT-qPCR primers were designed using Primer Premier 5.0 software (Supplementary Table 3). We chose the tubulin (No. AB239680.1) served as the reference gene for RT-qPCR analysis (Wu T. et al., 2012). The RT-qPCR was performed with a CFX96 Touch™ Real-Time PCR Detection System (BIO-RAD), each sample was subjected to three biological replicates. The relative changes in gene expression levels were calculated using the 2^–ΔΔ^ ^CT^ method (Livak and Schmittgen, 2001).

### Subcellular Localization of *PbBGLU1, PbBGLU15*, and *PbBGLU16*

The three genes RT-PCR primers were designed using Primer Premier 5.0 software (Supplementary Table 4). The full-length CDS of *PbBGLU1*, *PbBGLU15*, and *PbBGLU16* without stop codon were cloned based on genomic information and constructed into pCAMBIA1305 vectors (Clontech, Beijing, country-region China) between the *Xba*I and *Bam*HI (NEB) sites which have both CaMV35S promoter and GFP gene. After electroporation of these constructions into *Agrobacterium tumefaciens EHA105*, using pCAMBIA1305 vector as a negative control. The infection solution was injected into the epidermis of *Nicotiana tabacum* leaves, after culturing in the dark for 3 days, the glass slide was made and placed under the FV1200 laser confocal microscope (Olympus Corporation, Tokyo, Japan) to observe the distribution of fusion protein.

### *In situ* Hybridization

Segments of the 39 DAF fresh pear fruit were fixed in *in situ* hybridization fixed solution overnight. Pear pulp segments fixed: Take out the segment, wash, clean, put in the fixed fluid (DEPC) immediately to fix above 12 h. Dehydration: The segment is dehydrated by gradient alcohol, paraffin, embedding, and vacuum pumping in the dehydration process. Section: The paraffin is sliced through the slicer, the piece of the slicing machine, and the 62–degree oven roast for 2 h. Dewaxing and dehydration: Soak sections in 2 changes of xylene, 15 min each. Dehydrate in 2 changes of pure ethanol for 5 min each. Then, followed respectively by dehydrating in gradient ethanol of 85 and 75% ethanol 5 min each. Wash in DEPC dilution. Digestion: Mark the objective tissue with a liquid blocker pen, according to the characteristics of tissues, add proteinase K (20 ug/ml) working solution to cover objectives and incubate at 37°C for 22 min. Washing with pure water, then wash three times with PBS (pH 7.4) in a rocker device, 5 min each. Hybridization: Discard the pre-hybridization solution, add the probe hybridization solution, concentration, and incubate the section in a humidity chamber and hybridize overnight. Add the Alkaline Phosphatase-conjugated IgG Fraction Monoclonal Mouse Anti-Digoxin Antibody: (anti-DIG-AP): Remove the blocking solution and add anti-DIG-HRP. Incubate at 37°C for 40 min, then wash sections in TBS four times for 5 min each. NBT/BCIP developing: dry sections slightly, add fresh prepared NBT/BCIP (Thermo Scientific 1-Step NBT/BCIP) chromogenic reagent to marked tissue. Manage reaction time by observing in microscopy until positive expression appears shows blue-purple. Then stop developing reaction by a wash in running tap water. The probe sequence was unique to the PbBGLU1 and PbBGLU16 locus (Supplementary Table 4) and resulted in a single hit when used as a quarry to BLAST the Chinese white pear genome. *In situ* hybridization probes were synthesized by Sangon Bio Shang hai and labeled with Digoxigenin.

### Construction of PbBGLUs Expression Vector and Induced Purification of Recombinant Protein

The RT-PCR primers were designed using Primer Premier 5.0 software (Supplementary Table 4). The full-length coding sequence of *PbBGLU1* and *PbBGLU16* were cloned and then expressed in pGEX4T-1 vector (GE Healthcare, Chicago, IL, United States) between the SamI and *Not*I (NEB) sites. Two recombinant pGEX4T-1-PbBGLUs were transformed into *Escherichia coli* BL21 (DE3) Competent Cells. Add *Escherichia coli* BL21 (DE3) cells carrying pGEX4T-1-PbBGLU1 and pGEX4T-1-PbBGLU16 plasmid to 100 ml of LB liquid containing ampicillin for shaking. Expand the culture to an *OD* value of about 0.6, add isopropyl-β-D-thioacetamide IPTG to a final concentration of 1 mM/L to induce the fusion expression of the recombinant protein, culture with shaking at 16°C for 24 h, and collect the cells by centrifugation at 4°C. The centrifuged cells were suspended in PBS (pH 7.2–7.4) buffer, using an ultrasonic cell disruptor for disruption, then centrifuge to take the supernatant and discard the pellet. Using affinity chromatography resin with GST tag GST⋅ Bind™ resin (Novagen) to Purify protein. The Elution Buffer (pH 8.0) for GST-Sefinose (TM) Resin (containing reducing glutathione reagent at a concentration of 5 mM) Eluent was used for elution.

### Recombinant Protein Enzyme Assays

To analyze the activity of recombinant protein GST-PbBGLU1 and GST-PbBGLU16, 10 μg of purified protein was incubated at 35°C with 20 μL buffer (50 mM MgSO4, 200 mM KCL, 100 mM PBS pH 7.2–7.4) and 1 mM substrates (coniferin and syringin). The water was added to a final volume of 50 μL. After 1 h of reaction, add 50 μL methanol termination reaction. All reactions were supplemented with 0.1% (V/V) β- Mercaptoethanol. Then the reaction results were analyzed by HPLC.

To calculate the *K*_*m*_, *V*_*max*_, and *k*_*cat*_ value of the recombinant protein, under the same original reaction conditions, the substrates (coniferin and syringin) were set to 10 concentrations (0.025, 0.05, 0.075, 0.1, 0.15, 0.2, 0.3, 0.4, 0.5, and 0.6 mM). Then the product concentration C is calculated by an external standard method, and then the reaction rate V is further calculated. Finally, use the double reciprocal method to plot to calculate *K*_*m*_ and *V*_*max*_.

### Genetic Transformation of *Arabidopsis* and Transient Transformation of Pear Fruit

The RT-PCR primers were designed using Primer Premier 5.0 software (Supplementary Table 4). The full-length coding sequence of *PbBGLU1* and *PbBGLU16* were cloned from “Dangshan Su” pear first-strand complementary DNA (cDNA) with PrimeSTAR^®^ Max DNA Polymerase (Takara, Japan). The fragment was cloned into pCAMBIA1301-35S binary vectors between the *Bam*HI and *Xba*I (NEB) sites and used in plant transformation. Wild type and *Arabidopsis At1g61810 BGLU45-2* mutant were used for transformation with *A. tumefaciens* GV3101 carrying the above binary plasmid using the floral dip method.

The full-length coding fragment was cloned into pCAMBIA1301-35S binary vectors and used in the transient injection of pear fruit overexpression. Hairpin constructs were based on the pRNAiDE001 vector (Heneghan et al., 2007; Eastwood et al., 2008) where self-complementary sense (PbBGLU1: 905-1220bP; PbBGLU16: 832-1183bP) and antisense sequences are separated by a non-functional sequence (loop), then construct this fragment into the pCAMBIA1301 vector with the 35S promoter (Supplementary Figures 1, 2). Pear trees (40-year-old) and 39 days after flowering young fruits were selected for transient injection. The constructed pCAMBIA1301-35s binary vectors of *PbBGLUs* overexpression and *PbBGLUs* RNA interference gene were injected on the left of the fruit, and the empty pCAMBIA1301-35s binary vectors were injected on the right for control. The method followed a previously reported protocol (Zhang et al., 2021) nine fruits were injected with each construct in an experiment that was repeated three times independently. After 10 days, the transient injected fruits were picked up.

### Lignin Determination and Histochemical Staining of *Arabidopsis* and Pear Fruits

We collected the *Arabidopsis* plants for approximately 6 weeks, removed the leaves, and dried them in an oven at 65°C for 48 h. The lignin content of the *Arabidopsis* plants was estimated following the method of Anderson et al. (Anderson et al., 2015).

We obtained the inflorescence stem segments (young stem regions) of the transgenic and wild-type *Arabidopsis* plants for approximately 6 weeks. The samples were placed in a solution containing 95% methanol, 70% (v/v) ethanol and glacial acetic acid for 12 h and embedded in paraffin for sectioning with the pathology slicer (RM 2018). Plant tissue sections were stained following standard phloroglucinol staining protocols (Pradhan and Loqué, 2014).

After the transiently injected pear fruits was brought back to the laboratory, samples 5 g pulp from 2.0 mm under the peel to 0.5 mm outside the core, was collected and frozen at –80°C for 24 h, homogenized with a high-speed homogenizer for 3 min, rotating speed was 20,000 RPM/min, added water and stood for a while, then poured out the upper suspension, repeated several times until the upper layer was clear, then filtered and dried stone cells were weighed, repeated for 3 times, stone cell content = measured stone cell dry weight/5 × 100%. The lignin content was measured using the Klason method (Raiskila et al., 2007). A small amount (0.2 g) of stone cells was extracted with 15 ml of 72% H_2_SO4 at 30°C for 1 h, combined with 115 ml of distilled water, and boiled for 1 h. The volume was kept constant during boiling. The liquid mixture was filtered and the residue was rinsed with 500 ml of hot water, air-dried and weighed. The lignin content was shown as a percentage (calculated lignin content/dry weight of stone cells × 100%) (Zhang et al., 2021).

### Targeted Metabolite Determination

HPLC (Thermo Scientific UltiMate 3000 HPLC) analysis was carried out using a Columbus (Thermo Scientific 5-μm C18 column; 250×4.60 mm). Acetonitrile (solvent A) and H_2_O (solvent B) with gradient of 10–30% acetonitrile in water (all solutions contained 0.1% trifluoroacetic acid) with a flow rate at 1 ml min^–1^ over 35 min was used. Each peak on the chromatogram was scanned between 200 and 400 nm (photodiode array profile) and was integrated at 264, 280, and 324 nm. The data were acquired and analyzed using the software ChromQuest version. Extraction of tissue was carried out as described in Lanot et al. (2006).

## Results

### Identification of *BGLU* Genes in *Pyrus bretschneideri*

The BGLU family conserved domain (Pfam: PF00232) was used as a target query sequence, and the corresponding hidden Markov model was obtained from the Pfam website (see text footnote 2) which was used to identify the *BGLU* members. SMART (see text footnote 3) was used to check whether there are characteristic domains, with the redundant and repeated sequences ultimately removed. As a result, we identified 50 *BGLU* genes from Chinese white pear (*P. bretschneideri*). The detailed information on the gene ID, gene name, chromosomal location, protein structure, and characteristics of the corresponding BGLU proteins are listed in Supplementary Table 1.

### Phylogenetic Analyses of the Conserved Motif and the Exon–Intron Structure of *BGLU* Genes in Chinese White Pear

**FIGURE 1:**
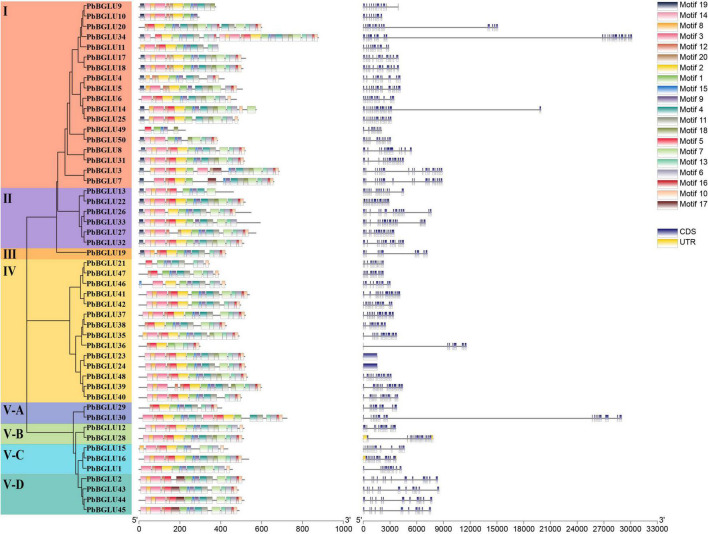
Phylogeny, conserved motifs and exon-intron structure of *BGLU* genes in pear. MEME tools were used to identify motifs. Different colors represent different motifs. GSDS online website was used to generate structure. The legend is located at the bottom right of the figure. Scale represents the length of the DNA sequence.

Inclusive of all *PbBGLU* genes, a phylogenetic tree was constructed with MEGA 7.0 software using the neighbor-joining (NJ) method (bootstraps = 1,000), which divided them into 8 groups (I, II, III, IV, V-A, V-B, V-C, V-D) with supported bootstrap values (Figure 1). We used MEME online software to analyze the amino acid sequence of *PbBGLU* genes and identified 20 motifs among all the sequences (Supplementary Table 2 and Figure 1). All the *PbBGLU*s contain motif1 (CTGGBSATEPYJVAHHQLLAHAAAVKLYREKYQA), and only two genes do not contain motif9 (WFEPASES KEDKAAALRALDF), suggesting that motif1 and motif9 are conserved gene motifs of *PbBGLU*s family. Only group I, group II, and group III genes contain motif19, whereas the other groups do not, and no members of group V-D contain motif2, it is speculated that these motifs may have special significance. Previous studies implied that gene structural diversity can lead to the evolution of multi-gene families (Dong et al., 2019). To better characterize and understand the structural diversity of the *PbBGLU*s, gene exon-intron analysis was carried out (Figure 1). Notably, *PbBGLU23* and *PbBGLU24* have only one exon, whereas the others contain 7 or more exons; *PbBGLU34* contains the most exons (21), and most genes had between 10 and 15 exons. These results indicate the possible occurrence of alternative splicing during gene evolution, which in turn leads to functional differences between these closely related *PbBGLU*s (Cao et al., 2017; Dong et al., 2019).

**FIGURE 2:**
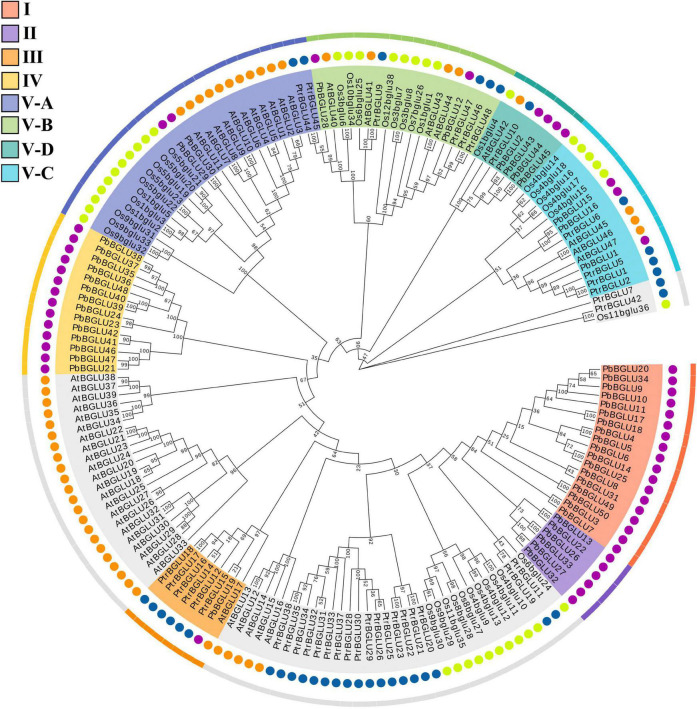
Phylogenetic analysis of *BGLU*s from *Pyrus bretschneideri*, *Arabidopsis thaliana*, *Oryza sative*, and *poplar*. The neighbor-joining (NJ) method was used to construct the phylogenetic tree. Pb: *Pyrus bretschneideri*; Os: *Oryza sative*; Ptr: *Poplar* L.; At: *Arabidopsis thaliana*.

To further understand the evolutionary relationship and related functions of *PbBGLU*s, a phylogenetic tree comprising *BGLU*s from *A. thaliana* (Xu et al., 2004), *O. sative* (Opassiri et al., 2006), *poplar* (Tsuyama and Takabe, 2015), and *P. bretschneideri* was constructed (Figure 2). We marked the position of the eight groups in pear on this phylogenetic tree and found that group I, group II, and group IV contained only *PbBGLU*s but not *AtBGLU*s, *Osbglu*s, and *PtrBGLU*s, indicating that some changes occurred among the *BGLU*s of different species during the evolutionary process (Cao et al., 2016a,b). *Arabidopsis AtBGLU17* (Roepke et al., 2017) was related to the use of *Arabidopsis* flavonoids which was clustered together with *PbBGLU19* (group III), and it is speculated that *PbBGLU19* might be involved in the hydrolysis of flavonoid glycosides in pear. *PbBGLU* group V-D clustered together with *AtBGLU42*, and which encodes an MYB72-dependent key regulator of rhizobacterium-induced systemic resistance and modulates iron deficiency responses in *Arabidopsis* roots (Zamioudis et al., 2014). *AtBGLU1-6*, which plays a role in the accumulation of flavonoids in *Arabidopsis* (Ishihara et al., 2016), clustered together with *PbBGLU* group V-A genes, speculating that *PbBGLU29* and *PbBGLU30* may have similar functions in pear. The functions of *AtBGLU45* and *AtBGLU46* in *Arabidopsis* (Escamilla-Treviño et al., 2006; Chapelle et al., 2012); *Os4BGlu14*, *Os4BGlu16*, and *Os4BGlu18* in *O. sative* (Baiya et al., 2014; Baiya et al., 2018); *PtrBGLU6* in *poplar* (Tsuyama and Takabe, 2015) have been clarified, and their products catalyze the hydrolysis of monolignol glucosides during lignification. The three genes (*PbBGLU1*, *PbBGLU15*, and *PbBGLU16*) in *PbBGLU* group V-C clustered together with these functional genes, speculating that *PbBGLU1*, *PbBGLU15*, and *PbBGLU16* may be able to affect lignification by catalyzing the hydrolysis of monolignol glucosides in pear fruit, which in turn affects the development of pear stone cells.

### Expression Characteristics of Chinese White Pear BGLU Genes

**FIGURE 3:**
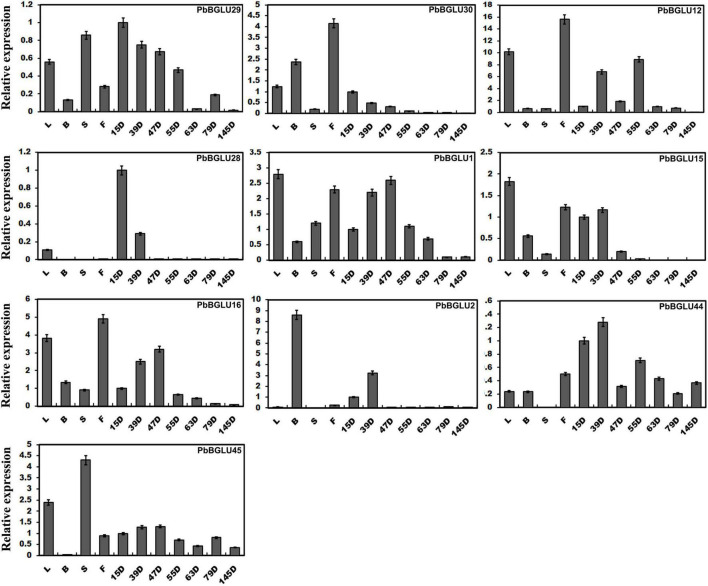
Expression levels of PbBGLU genes in different plant tissues. 15D, 39D, 47D, 55D, 63D, 79D, and 145D correspond to seven different developmental stages (days after flowering) of pear fruit. In addition, leaf, bud, stem, and flower were represented by L, B, S, and F, respectively. The value on the left Y-axis indicates the relative gene expression levels.

We constructed a phylogenetic tree comprising *BGLU*s genes from *A. thaliana* (Xu et al., 2004), *O. sative* (Opassiri et al., 2006), *poplar* (Tsuyama and Takabe, 2015), and *P. bretschneideri*, and found that *PbBGLU1*, *PbBGLU15*, and *PbBGLU16* in the group V-C *PbBGLU*s of pear clustered together with *AtBGLU45*, *AtBGLU46, Os4BGlu14*, *Os4BGlu16*, *Os4BGlu18*, and *PtrBGLU6* functional genes, which can hydrolyze monolignol glucosides during lignification (Escamilla-Treviño et al., 2006; Chapelle et al., 2012; Baiya et al., 2014; Tsuyama and Takabe, 2015; Baiya et al., 2018). To verify whether *PbBGLU1*, *PbBGLU15*, and *PbBGLU16* have similar functions, we selected all *PbBGLU* genes in group V (a total of 11 *PbBGLU*s) for qRT-PCR analysis. The expression profiles of these genes in different tissues of pear (leaves, buds, stems, flowers) and pear fruit at different developmental stages (15, 39, 47, 55, 63, 79, and 145 days after flowering) were also investigated (Figure 3). It found that *PbBGLU43* was not expressed in different tissue parts or at different developmental stages of the fruit. The expression levels of the other ten *PbBGLU*s were generally high in leaves, buds, flowers, and young fruits (15, 39, 47, and 55 days after flowering). This result was consistent with those of previous research showing that β-glucosidase can be found mainly in young vegetative parts (Kristoffersen et al., 2000; Biely et al., 2003). The expression levels of the *PbBGLU1*, *PbBGLU15*, and *PbBGLU16* genes were relatively high in the leaves and flowers as compared to buds and stems. All three genes were expressed at different stages of fruit development, and their expression showed a trend of first increasing then decreasing, with a similar trend of the content for lignin and stone cells in “Dangshan Su” pear fruit (Cai et al., 2010; Jin et al., 2013). The expression patterns of these three genes were also similar to those of the key genes involved in the regulation of the lignin synthesis pathway (Xie et al., 2013). These results suggested that the three genes (*PbBGLU1*, *PbBGLU15*, and *PbBGLU16*) in the group V-C *PbBGLU*s might be functional genes involved in the hydrolysis of monolignol glucosides in the pear lignin synthesis pathway.

### Subcellular Localization of PbBGLU1, PbBGLU15, and PbBGLU16

**FIGURE 4:**
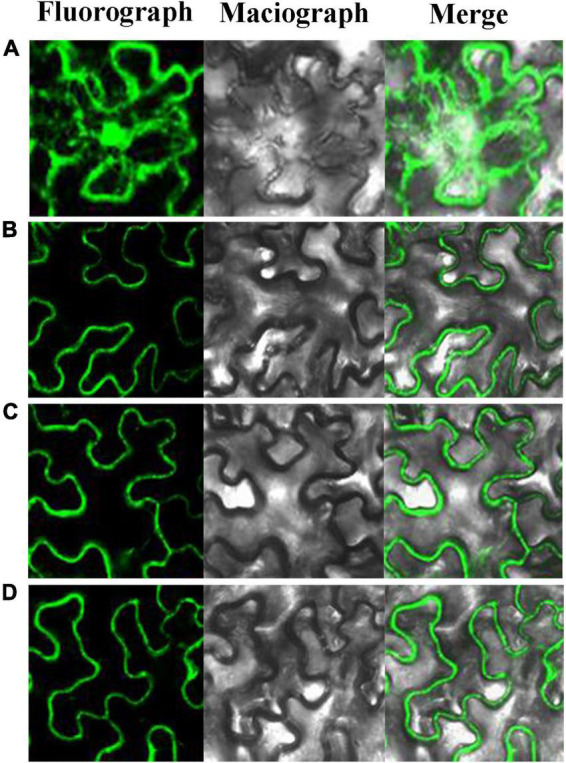
Subcellular localization of PbBGLU1-GFP, PbBGLU15-GFP, and PbBGLU16-GFP fusion protein. **(A)** GFP control; **(B)** Subcellular localization of PbBGLU1-GFP fusion protein; **(C)** Subcellular localization of PbBGLU15-GFP fusion protein; **(D)** Subcellular localization of PbBGLU16-GFP fusion protein.

As early as 1995, it found that *Pinus contorta* β-glucosidase activity occurred in the cell wall of secondary xylem tissue (Dharmawardhana et al., 1995). *AtBGLU45* and *AtBGLU46* of *A. thaliana* were also expressed in the cell wall (Chapelle et al., 2012). The rice Os4BGlu16 tagged with a C-terminal GFP was transiently expressed in tobacco leaf epithelial cells, which revealed that the GFP signal was exclusively localized to the plasma membrane and extracellular space in both intact and plasmolyzed cells (Baiya et al., 2018). It is consistent with apoplastic or cell wall localization. In this study, to explore the subcellular localization of *PbBGLU1*, *PbBGLU15*, and *PbBGLU16*, PbBGLU-GFP expression vectors were constructed and transformed into *N. tabacum*. As shown in Figure 4, green fluorescence signals from the expressed PbBGLU1-GFP, PbBGLU15-GFP, and PbBGLU16-GFP fusion constructs were specifically distributed in the extracellular region.

### *In situ* Hybridization Analysis of *PbBGLU1* and *PbBGLU16* in 39 DAF Pear Fruit

**FIGURE 5:**
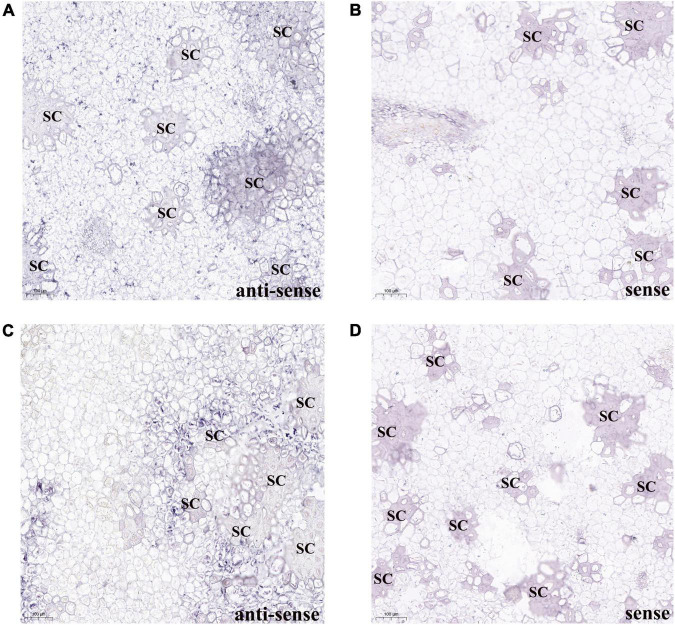
RNA *in situ* hybridization illustrating PbBGLU1 and PbBGLU16 transcript localization in 39 DAF (day after flower) pear fruit. **(A,B)** PbBGLU1 RNA *in situ* hybridization in 39 DAF (day after flower) pear fruit. **(C,D)** PbBGLU16 RNA *in situ* hybridization in 39 DAF (day after flower) pear fruit; Sections were taken through the flesh and probed with anti-sense **(A,C)** and sense probes **(B,D)** and imaged at 10 magnification. The positive expression of BCIP/NBT was blue-purple; the sense probes is for negative control; the bar = 100 μm; SC, stone cells.

To confirm the *PbBGLU1* and *PbBGLU16* gene expression associated with those stone cells undergoing lignin deposition and secondary wall thickening, we carried out RNA *in situ* hybridization (Wu and Wagner, 2012) on 39 DAF (days after flowering) pear fruit. The results of *PbBGLU1* transcripts *in situ* hybridization with *PbBGLU1* anti-sense probe showed that *PbBGLU1* transcripts were not only localized to some pulp cell walls, lignin deposition and stone cell areas of a pear fruit, there was also a small amount of enrichment in normal pear flesh cells (Figure 5A). This result shows that *PbBGLU1* is not only involved in lignin biosynthesis but also may be involved in other metabolic activities during pear fruit development. However, *PbBGLU16* transcripts were only enriched in lignin deposition and stone cell areas of pear fruit (Figure 5C), indicating that it may be a specific gene for lignin synthesis in pear fruit. Hybridization with control *PbBGLU1* and *PbBGLU16* sense probe showed no labeling as expected (Figures 5B,D).

### Enzymatic Assays of Recombinant PbBGLU1 and PbBGLU16 *in vitro*

**FIGURE 6:**
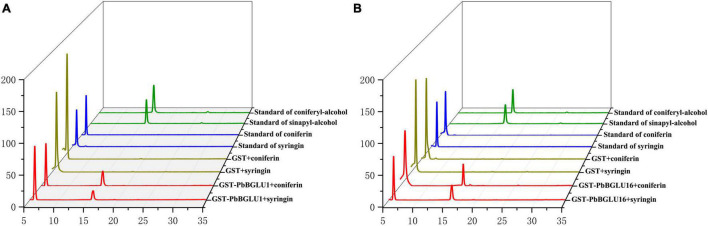
Enzyme activity of GST-PbBGLU1 and GST-PbBGLU16 recombinant protein toward coniferin and syringin. **(A)** HPLC analysis of standards and reaction products of GST-PbBGLU1 recombinant protein. **(B)** HPLC analysis of standards and reaction products of GST-PbBGLU16 recombinant protein. Reaction conditions: 10 μg of purified protein was incubated at 35°C with 20 μL buffer (50 mM MgSO4, 200 mM KCL, 100 mM PBS pH 7.2–7.4) and 1 mM substrates (coniferin and syringin), the water was added to a final volume of 50 μL. After 1 h of reaction, add 50 μL methanol termination reaction.

*PbBGLU1* and *PbBGLU16* were cloned into pGEX4T-1 vector, and then the constructed vector was induced and expressed in BL21 (ED3) *E. coli*. A target protein GST-PbBGLU1 with a weight of 78.07 kDa and another target protein GST-PbBGLU16 with a weight of 87.23 kDa obtained (GST: 26kDa; *PbBGLU1*: 52.07 kDa; *PbBGLU16*: 61.23 kDa). The sodium dodecyl sulfate-polyacrylamide gel electrophoresis (SDS-PAGE) was used to analysis and verification (Supplementary Figure 3). To verify whether the recombinant protein has the ability to glycosylate the monolignol glucosides coniferin, syringin. We added recombinant protein, coniferin, and syringin and buffer in the certain reaction system. After the reaction for a while, methanol was added to terminate the reaction and then put into the HPLC for detection. The standard of coniferyl alcohol, sinapyl alcohol, coniferin and syringin were put into the HPLC for reference (Figures 6A,B). The results showed that after the recombinant protein was added, two peaks of the substrate (coniferin and syringin) and product (coniferyl alcohol and sinapyl alcohol) were detected by HPLC. This phenomenon indicates that the recombinant protein of GST-PbBGLU1 and GST-PbBGLU16 were active against both substrates (coniferin and syringin). To calculate the kinetic parameters of the recombinant protein for coniferin and syringin enzyme, 10 substrates with different concentrations were set in this study. The *K*_*m*_ values, *V*_*max*_ and *k*_*cat*_ of GST-PbBGLU1 and GST-PbBGLU16 were calculated through the double reciprocal method. The results are shown in Supplementary Figure 4 and Table 1. Compared with syringin, GST-PbBGLU1 and GST-PbBGLU16 had a stronger activity and faster catalytic efficiency for coniferin. In addition, compared with GST-PbBGLU1, GST-PbBGLU16 shows higher catalytic activity and catalytic efficiency for the two substrates.

**TABLE 1:** Enzyme kinetics of PbBGLU1 and PbBGLU16.

Protein	Substrates	*K*_*m*_ (μM)	*V*_*max*_ (μM/min⋅mg)	*k*_*cat*_ (S^–1^)	*k*_*cat*_/*K*_*m*_ (mM^–1^S^–1^)
pGEX4T-1-PbBGLU1	coniferin	102.59 ± 3.89	1.76 ± 0.04	0.23 ± 0.01	2.24 ± 0.02
	syringin	100.77 ± 6.51	1.57 ± 0.05	0.20 ± 0.02	1.98 ± 0.07
pGEX4T-1-PbBGLU16	coniferin	98.01 ± 4.98	3.73 ± 0.10	0.54 ± 0.01	5.52 ± 0.17
	syringin	119.48 ± 8.17	2.46 ± 0.09	0.36 ± 0.01	3.02 ± 0.12

### Analysis of Transgenic *Arabidopsi*s With *PbBGLU1* and *PbBGLU16* Gene

**FIGURE 7:**
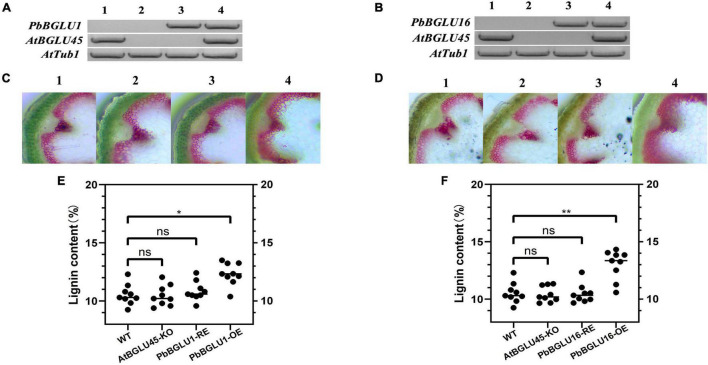
The lignin content in inflorescence stems of Arabidopsis. **(A)** PCR analysis of *Arabidopsis thaliana* wild type **(A-1,B-1)**; *AtBGLU-45* mutant **(A-2,B-2)**; *AtBGLU-45* mutant lines rescued by *PbBGLU1* and *PbBGLU16* **(A-3,B-3)**; *PbBGLU1* and *PbBGLU16* overexpressing *Arabidopsis* **(A-4,B-4)**. **(C,D)** Cross-sections of the inflorescence stem were stained with phloroglucinol–HCl; **(C-1,D-1)** Wild-type (WT) plants; **(C-2,D-2)** *BGLU45* knock out mutant plants; **(C-3,D-3)** *PbBGLU1* and *PbBGLU16* rescue plants; **(C-4,D-4)** Over-expression *PbBGLU1* and *PbBGLU16* plants. **(E,F)** Measurement of the lignin content in inflorescence stems of *Arabidopsis thaliana*. The bar = 100 μm. (* *P* < 0.05, ** *P* < 0.01). KO, knock out; RE, rescue; OE, over expression.

The *PbBGLU1* and *PbBGLU16* were transferred into *Arabidopsis BGLU-45* mutants (*PbBGLU1-RE* and *PbBGLU16-RE*) and wild-type *Arabidopsis* (*PbBGLU1-OE* and *PbBGLU16-OE*), reverse transcription PCR validation of *Arabidopsis* is shown in Figures 7A,B. To directly observe the *in situ* distribution of lignin in inflorescence stems of these *Arabidopsis* plants, we performed the Wiesner (phloroglucinol-HCl) histochemical staining (Pradhan and Loqué, 2014) of inflorescence stems of *Arabidopsis* plants. The Wiesner staining results indicated that the xylem and intravascular fibers in the stems of *Arabidopsis BGLU-45* mutants showed no increase to any extent compared with wild-type *Arabidopsis* (Figures 7C-2,D-2), this result is consistent with the results of previous studies (Chapelle et al., 2012). Then, we observed the stained sections of *PbBGLU1-OE* and *PbBGLU16-OE Arabidopsis* and the Wiesner staining results indicated that the xylem and intravascular fibers in the stems of *Arabidopsis PbBGLU1-OE* and *PbBGLU16-OE* showed stronger phloroglucinol staining compared to the wild-type plants (Figures 7C-4,D-4). According to the staining results, we can also find that *PbBGLU16-OE* xylem and intravascular fibers *Arabidopsis* increase more strongly than *PbBGLU1-OE Arabidopsis*. Subsequently, we measured the lignin content of wild-type, *Arabidopsis BGLU-45* mutant, *PbBGLU1-RE*, and *PbBGLU16-RE*, *PbBGLU1-OE*, and *PbBGLU16-OE Arabidopsis* by acetyl bromide method (Anderson et al., 2015; Figures 7E,F). It found that the lignin content of *Arabidopsis BGLU-45* mutant, *PbBGLU1-RE* and *PbBGLU16-RE* was no change than that of wild-type. However, compared with wild-type *Arabidopsis* the overexpression plants lignin increased in varying degrees, the effect of *PbBGLU16* on the increase of lignin in *Arabidopsis* is greater than that of *PbBGLU1*.

### Targeted Metabolite Determination

In order to further explore how *BGLU-45* mutants and overexpression of *PbBGLU1* and *PbBGLU16* affect the lignin of *A. thaliana*, we measured the *A. thaliana* contents of coniferin and syringin by HPLC. The result showed that coniferin and syringin increased significantly in *BGLU-45* mutant compared with the wild type (Table 2), but the lignin did not increase. This phenomenon occurred probably because coniferin and syringin are not precursors of lignin synthesis and usually exist in vacuoles as transport or storage forms. This is also probably the reason why there is no significant change in *Arabidopsis* lignin after knocking out *BGLU45* in previous research (Chapelle et al., 2012; Baiya et al., 2018). Then, the contents of coniferin and syringin rising phenomenon have been restored through transferred *PbBGLU1* and *PbBGLU16* into *Arabidopsis BGLU-45* mutant (Table 2). However, the lignin monolignol glycosides of transgenic *Arabidopsis* after overexpression of *PbBGLU1* and *PbBGLU16* decreased to some extent, but the decrease was not obvious, it may be because the decrease of lignin monolignol glycosides will increase the expression of *UGT*.

**TABLE 2:** HPLC analysis of soluble monolignol glucosides metabolites in *Arabidopsis*.

Materials	Genotypes	Coniferin (μmol/5 g)	Syringin (μmol/5 g)
Inflorescence stems	WT	0.135 ± 0.03	0.062 ± 0.02
	BGLU45-KO	0.251 ± 0.01	0.104 ± 0.02
	PbBGLU1-RE	0.105 ± 0.01	0.051 ± 0.01
	PbBGLU16-RE	0.126 ± 0.04	0.058 ± 0.03
	PbBGLU1-OE	0.132 ± 0.02	0.059 ± 0.03
	PbBGLU16-OE	0.138 ± 0.03	0.063 ± 0.03

### Transient Transformation of *PbBGLU1* and *PbBGLU16*, *PbBGLU1-RNAi* and *PbBGLU16-RNAi* in Pear Fruit

**FIGURE 8:**
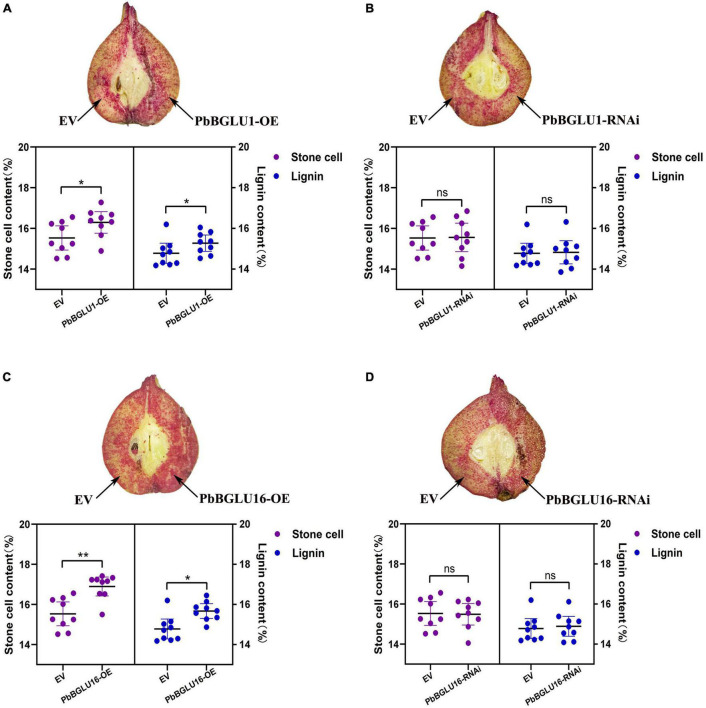
Transient assays using PbBGLU1 and PbBGLU16 silencing and overexpression constructs in “Dangshansuli” fruit at 39 days after flowering (DAF). Images were taken at 10 days after agro-infiltration. Cross-sections of PhBGLU1, PbBGL.U16 overexpression **(A,C)** and silencing **(B,D)** pear fruits were stained with phloroglucinol–HCI. Lignin and stone cell contents in PbBGLU1, PbBGLU16 overexpression **(A,C)** and silencing **(B,D)** flesh tissue around the infiltration sites, more than three fruits were injected with each construct in an experiment that was repeated three times. EV, empty vector; (* *P* < 0.05, ** *P* < 0.01).

Since the stable genetic transformation system of pears is in its infancy, it is difficult to perform a functional analysis of the *PbBGLU1* and *PbBGLU16* in pears. Therefore, we chose the method of transient expression by injection for analysis. We injected transiently the *PbBGLU1*, *PbBGLU16*, and *PbBGLU1* and *PbBGLU16* RNA interference in the 39 DAF (days after flowering) fruit of the pear tree. By Wiesner (phloroglucinol-HCl) histochemical staining (Pradhan and Loqué, 2014), the results show (Figures 8B,D) that when *PbBGLU1* and *PbBGLU16* were silenced by RNA interference, the change of pear lignin and stone cells was not obvious compared with the empty vector injection. But after *PbBGLU1* and *PbBGLU16* gene were overexpressed in the 39 DAF pear fruit, it found that the lignin and stone cells increased compared with the empty vector injection control in the pear fruit (Figures 8A,C). Moreover, compared with the transient expression of *PbBGLU1*, *PbBGLU16* had a stronger effect on the lignin and expression cells of pear fruit. To explore the specific effects of overexpression and RNAi of *PbBGLU1* and *PbBGLU16* on stone cells and lignin of pear fruit, we measured the contents of stone cells and lignin in pear fruit after empty vector, overexpression, and RNAi injection. The results are shown in Figures 8B,D which indicate that when *PbBGLU1* and *PbBGLU16* were silenced, the contents of stone cells and lignin in pear fruit did not change compared with empty vector injection. However, in pear fruits with transient overexpression of *PbBGLU1*, the contents of lignin and stone cells in pear fruits were significantly higher (0.01 < *P* < 0.05) than those in pear fruits with empty vector injection. After transient expression of *PbBGLU16*, lignin in pear fruit increased significantly compared with the control group (0.01 < *P* < 0.05), while the content of stone cells in pear fruit injected with *PbBGLU16* showed a very significant difference (*P* < 0.01) compared with the control group.

## Discussion

In plants, glycoside hydrolase (GH) family 1 β-glycosidases are believed to play an important role in many diverse processes, including chemical defense against herbivory, hydrolysis of cell wall-derived oligosaccharides during germination, control of active phytohormone levels, and lignification (Xu et al., 2004; Lee et al., 2006; Ahn et al., 2010; Xu et al., 2012; Zhou et al., 2012; Miyahara et al., 2013).

The complete sets of members of the GH1 BGLU genes family have been identified only in a few species. We identified a total 50 *BGLUs* in pear. The number was similar to that in *Arabidopsis* (48) (Xu et al., 2004) and poplar (48) (Tsuyama and Takabe, 2015), but more than that in rice (34) (Opassiri et al., 2006) and maize (26) (Gomez-Anduro et al., 2011). Previous studies have shown that the role of monolignol glucosides was necessary for transmembrane transport (Tsuyama and Takabe, 2015; Julien et al., 2016), monolignol glucosides form can increase the solubility and stability of monolignols, which is very beneficial for the transportation of lignin, ultimately to specific locations at the cell wall, after which point lignin begins to deglycosylate and polymerize (Liu, 2012). Many previous studies have shown that BGLU genes are localized to the cell wall and extracellular space (Dharmawardhana et al., 1995; Chapelle et al., 2012; Baiya et al., 2018). In this study, we found that green fluorescence signals from the expressed PbBGLU1-GFP, PbBGLU15-GFP, and PbBGLU16-GFP fusion constructs were specifically distributed in the extracellular region, which is consistent with previous research. Also, in our *in situ* hybridization analysis, it found that *PbBGLU1* and *PbBGLU16* transcripts were mainly located in lignin deposition and stone cell areas of pear fruit. This result further suggests that *PbBGLU1* and *PbBGLU16* may be functional genes in the process of lignin synthesis and stone cell development in pear.

The heterologous expression of *Arabidopsis* BGLU45 and BGLU46 genes in *Pichia pastoris* revealed that BGLU45 was highly specific for the three monolignol glucosides (coniferin, syringin, and p-coumaryl alcohol 4-O-b-D-glucoside). By contrast, BGLU46 has p-coumaryl alcohol glucoside as the preferred substrate, but it displays a broad specificity toward various substrates (Xu et al., 2004; Escamilla-Treviño et al., 2006). The *in vitro* substrate specificity of BGLU45 and BGLU46 and the expression of their genes in stems (Escamilla-Treviño et al., 2006) support the hypothesis that these enzymes are involved in the lignification of *Arabidopsis*. To further, verify the functions of *PbBGLU1* and *PbBGLU16*, we induced expression and purification recombinant protein, and selected coniferin and syringin as substrates (Dicotyledons mainly contain syringic units and guaiacyl unit lignin: G-S). Enzyme activity analysis showed that the two recombinant proteins could act on coniferin and syringin to deglycosylate them and produce coniferyl alcohol and sinapyl alcohol. Moreover, compared with GST-PbBGLU1 recombinant protein, GST-PbBGLU16 recombinant protein shows higher activity and catalytic efficiency for the two substrates.

In previous studies, the implication of monolignol glucosides in the lignification process has been repeatedly suggested (Boerjan et al., 2003). The hypothesis was that monolignol glucosides would be stored in the vacuole or transported from the cytosol to the cell. One of the most predominant questions about phenylpropanoid biosynthesis concerns the spatial distribution of both precursors and final products within the cells. Many phenylpropanoid molecules necessary for plant development and/or defense are also toxic and an important challenge for plants is how to successfully manage the production/storage of these potentially dangerous molecules (Väisänen et al., 2015). In previous studies, the knock-out of *BGLU-45* and *BGLU-46* in *Arabidopsis* can lead to a significant increase of monolignol glycosides, but the lignin content does not seem to change significantly (Chapelle et al., 2012; Baiya et al., 2018). We also obtained the same results in our experiment. After transferred *PbBGLU1* and *PbBGLU16* into *BGLU-*45 *Arabidopsis* mutant, we found that the rise of these two glycosides in *BGLU-*45 *Arabidopsis* mutant was some degree of recovery, which further proved the function of *PbBGLU1* and *PbBGLU16.* When *PbBGLU1* and *PbBGLU16* were overexpressed into *Arabidopsis*, the lignin content of *A. thaliana* was significantly increased, and the increasing degree of *PbBGLU16-OE* was greater than that of *PbBGLU1-OE*. The same result also occurred in the experiment of instantaneous injection in pear fruit, when we silenced *PbBGLU1* and *PbBGLU16* by RNA interference, there was no significant difference in the contents of lignin and stone cells between pear and the control group, but the contents of stone cells and lignin were significantly increased when *PbBGLU1* and *PbBGLU16* were transiently overexpressed, and PbBGLU16 has a greater effect on lignin and stone cells than PbBGLU1. A past study also showed that the expression of *BGLU45* and *BGLU46* was deregulated under various biological stresses, suggesting that the monolignol storage pool might be used under pathogen attack in the fabrication of “defense lignin” and/or phytoalexins, cell, and vacuole disruption following pathogen attack, insect/herbivore feeding and potentially severe abiotic stress (cell freezing, desiccation) or during programmed cell death (xylem vessel) would lead to contact between themonolignol glucosides and β-glucosidases at the cell wall where PRXs and/or LACs could rapidly polymerize the aglycone forms (Morant et al., 2008; Chapelle et al., 2012). This also proves β-glucosidase can directly or indirectly increase lignin. However, monolignol glucosides had not been considered a dead-end product and its content is still regulated by many parties. Monolignol glucosides had often been detected in gymnosperms and its content in the differentiating xylems of gymnosperms peaks at around the cambium and decreases as lignification progresses (Fukushima et al., 1997; Tsuyama and Takabe, 2015). The regulation mechanism of lignin transport is very complex, which needs to be explored continuously. In conclusion, this study screened and proved the function of β-glucosidase during the process of lignin synthesis and stone cells development in pear fruit, and laid a foundation for improving pear fruit quality at the molecular level.

## Conclusion

In order to analyze the BGLU β-glucosidases in the glycoside hydrolase family 1 (GH1) of pear, bioinformatics analysis, qRT-PCR, subcellular localization, *in situ* hybridization, enzyme activity analysis, the transformation of *PbBGLU1* and *PbBGLU16* into *Arabidopsis*, and transient injection *PbBGLU1* and *PbBGLU16* into pear fruit experiment were performed in this study. A total of 50 non-redundant BGLU family members were identified in Chinese white pears while three candidate genes *PbBGLU1, PbBGLU15*, and *PbBGLU16* were identified that might be involved in lignin synthesis. Subcellular localization showed that all three genes were located on the extracellular region. *In situ* hybridization analysis revealed that *PbBGLU1* and *PbBGLU16* were involved in lignin synthesis and stone cell development in pear fruit. Enzyme activity analysis showed that GST-PbBGLU1 and GST-PbBGLU16 had activity on coniferin and syringin. The transformation of *PbBGLU1* and *PbBGLU16* into *Arabidopsis* demonstrated that as compare with wild-type *Arabidopsis*, the overexpression plants lignin increased in varying degrees and the effect of *PbBGLU16* on the increase of lignin in *Arabidopsis* was greater than that of *PbBGLU1*. In pear fruits with transient overexpression of *PbBGLU1* and *PbBGLU16*, the contents of *PbBGLU1-OE* and *PbBGLU16-OE* lignin and stone cells in pear fruits were significantly higher than those in pear fruits with empty vector injection, the effect of *PbBGLU16* on the increase of lignin and stone cells was greater than that of *PbBGLU1*. In summary, the identification of 50 *BGLUs* and analysis of these two BGLUs gene functions in pear will help to improve the quality of pear fruit in the future. These results provide information that may facilitate further functional analyses of PbBGLU genes to elucidate their biological roles in pear.

## Data Availability Statement

The original contributions presented in the study are included in the article/Supplementary Material, further inquiries can be directed to the corresponding author.

## Author Contributions

YoC conceived and designed the experiments. HW, YiZ, and XF performed the experiments. FP, MM, YaZ, WH, YuZ, JL, and YuC analyzed the data. HW wrote the manuscript. All authors reviewed and approved the final submission.

## Conflict of Interest

The authors declare that the research was conducted in the absence of any commercial or financial relationships that could be construed as a potential conflict of interest.

## Publisher’s Note

All claims expressed in this article are solely those of the authors and do not necessarily represent those of their affiliated organizations, or those of the publisher, the editors and the reviewers. Any product that may be evaluated in this article, or claim that may be made by its manufacturer, is not guaranteed or endorsed by the publisher.

## Footnotes

1
http://gigadb.org/dataset/10008

2
https://pfam.xfam.org/

3
http://smart.embl.de/

4
http://web.expasy.org/compute_pi/

5
https://meme-suite.org/meme/doc/meme.html

6
http://gsds.cbi.pku.edu.cn

7
https://phytozome.jgi.doe.gov/pz/portal.html#

## Abbreviations

BGLU: β-glucosidase
GH1: glycoside hydrolase family 1
HMM: hidden Markov model
NJ: neighbor-joining
qRT-PCR: real-time PCR
ABC transporter: ATP-binding cassette transporter.
