# Supplementary material for: Analysis of the β-Glucosidase Family Reveals Genes Involved in the Lignification of Stone Cells in Chinese White Pear (Pyrus bretschneideri Rehd.)
Source: Front Plant Sci. 2022 May 10;13:852001. doi: 10.3389/fpls.2022.852001 (PMC9127867; doi:10.3389/fpls.2022.852001)
Supplement: Supplementary file 1 [file Data_Sheet_1.PDF]

Analysis of the β-glucosidase Family reveals Genes Involved in the Lignification of Stone Cells in Chinese White Pear (*Pyrus bretschneideri* Rehd.)

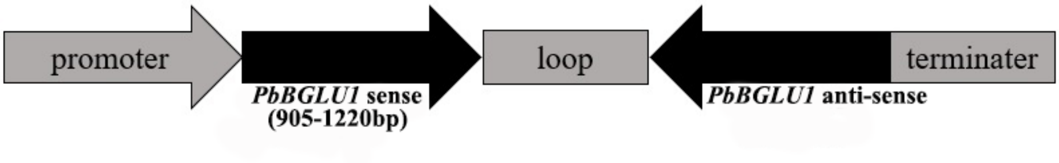

FIGURE S1. Construction of *PbBGLU1* RNA interference vector.

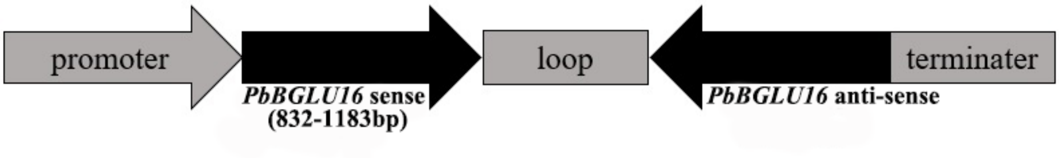

FIGURE S2. Construction of *PbBGLU16* RNA interference vector.

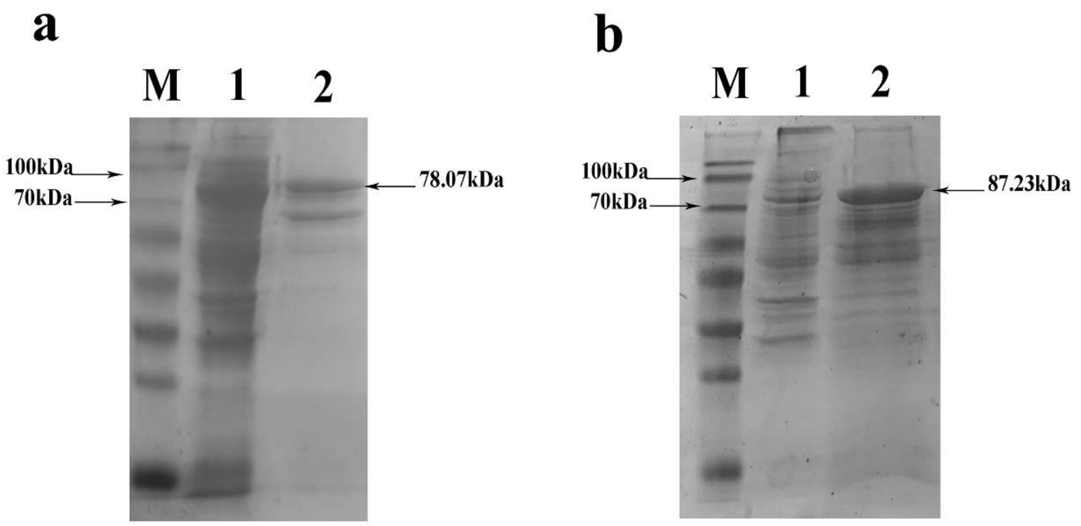

FIGURE S3. SDS-PAGE of protein fractions. a: M: marker; 1: soluble fractions after induced of GST-PbBGLU1; 2: purified fractions after induced of GST-PbBGLU1. b: M: marker; 1: soluble fractions after induced of GST-PbBGLU16; 2: purified fractions after induced of GST-PbBGLU16.

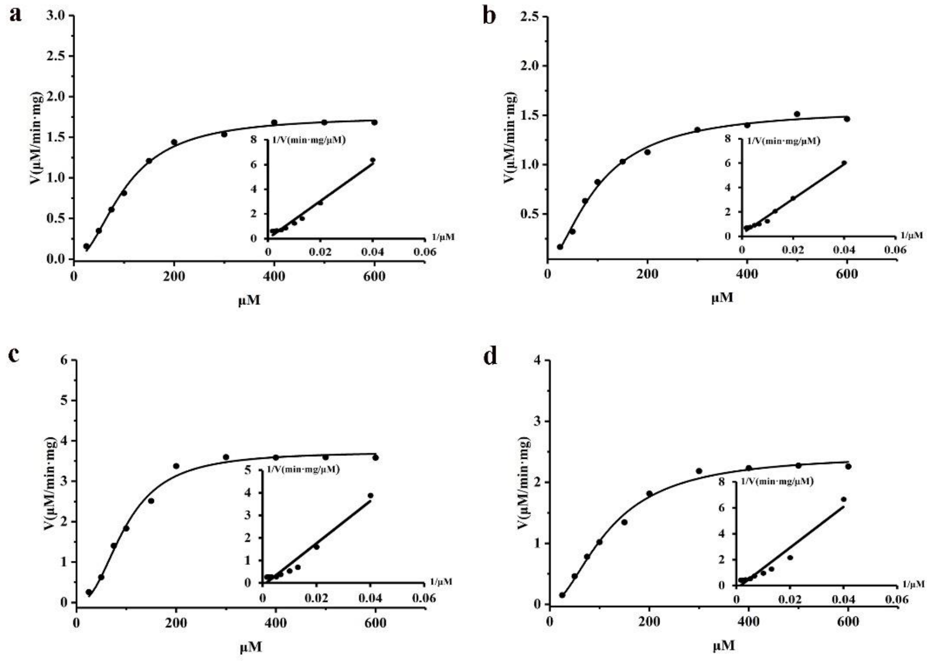

FIGURE S4. Initial reaction rate to concentration plots and reciprocal plots showing kinetics of GST-PbBGLU1 and GST-PbBGLU16 to coniferin and syringin substrates. a: GST-PbBGLU1 to coniferin substrates; b: GST-PbBGLU1 to syringin substrates; c: GST-PbBGLU16 to coniferin substrates; d: GST-PbBGLU16 to syringin substrates.
